# Induction of IL-22 protein and IL-22-producing cells in rainbow trout *Oncorhynchus mykiss*

**DOI:** 10.1016/j.dci.2019.103449

**Published:** 2019-12

**Authors:** Yehfang Hu, Yamila Carpio, Callum Scott, Ayham Alnabulsi, Abdo Alnabulsi, Tingyu Wang, Fuguo Liu, Milena Monte, Tiehui Wang, Christopher J. Secombes

**Affiliations:** a Scottish Fish Immunology Research Centre (SFIRC), School of Biological Sciences, University of Aberdeen, UK; b Centre of Genetic Engineering and Biotechnology, Havana, Cuba; c Vertebrate Antibodies Limited, Aberdeen, UK; d Department of Pathology, School of Medicine, Medical Sciences and Nutrition, University of Aberdeen, UK

**Keywords:** Rainbow trout, Interleukin-22, Transcript expression, Protein expression, Monoclonal antibody

## Abstract

IL-22 is a critical cytokine which is involved in modulating tissue responses during inflammation, and is produced mainly by T cells and innate leucocytes. In mammals, IL-22 is a key component in mucosal defences, tissue repair, epithelial cell survival and proliferation. In teleosts, IL-22 has been cloned and studied in several species, and the transcript is highly expressed in mucosal tissues and induced by pathogen associated molecular patterns (PAMPs), suggesting IL-22 also functions as an important component of the innate immune response in fish. To investigate these immune responses further, we have validated and characterised two monoclonal antibodies (mAbs) which were raised against two different peptide immunogens of salmonid IL-22. Our results show that both mAbs specifically react to their own peptide immunogens and recombinant IL-22, and are able to detect the induction of native protein expression after stimulation. In flow cytometry, an increase in IL-22 positive cells was detected after stimulation *in vitro* with cytokines and PAMPs and *in vivo* after bacterial challenge. The immunohistochemistry results showed that IL-22 is highly upregulated in the gills after challenge, both in cells within the gill filaments and in the interbranchial lymphoid tissue. Such results suggest IL-22 may have a role in triggering local antimicrobial defences in fish that may facilitate efficient microbial clearance. Hence monitoring IL-22 producing cells/protein secretion may provide an alternative mean to assess the effectiveness of mucosal vaccines.

## Highlights

• Two monoclonal antibodies (L7 & L8) were developed and characterised against rainbow trout IL-22.
• Both mAbs specifically react to their peptide immunogens, recombinant IL-22, and induced native IL-22 protein.
• IL-22 producing cells were detected by intracellular staining in flow cytometry following *in vitro* and *in vivo* stimulation.
• IL-22 transcript and protein expression is up-regulated in gills and PBLs in response to bacterial infection.
• IL-22+ cells were found in the gill epithelium of lamellae and interbranchial lymphoid tissue after bacterial infection.

## 1 Introduction

Interleukin (IL)-22 was originally identified as an IL-10-related T cell-derived inducible factor in 2000 (Dumoutier et al., 2000). It is a member of the IL-10 family that in mammals includes IL-10, IL-19, IL-20, IL-24, and IL-26, in addition to IL-22 (Dudakov et al., 2015). These molecules are themselves part of the class II α-helical cytokine family, that includes the interferons; type I, type II (IFN-γ) and type III (the λ-interferons IL-28A/B and IL-29) (Redmond et al., 2019). In mammals, IL-10 family cytokines are produced by both innate and adaptive immune cells, and exert essential functions to maintain tissue homeostasis during infection and inflammation (Ouyang and O'Garra, 2019). In the case of IL-22, it is produced mainly by lymphoid cells including αβ and γδ T cells, innate lymphoid cells (ILCs) and natural killer T (NKT) cells (Dudakov et al., 2015; Lanfranca et al., 2016). Three types of CD4^+^ T helper (Th) cells, Th1, Th17 and Th22 (Akdis et al., 2012; Rutz et al., 2013), as well as CD8^+^ T cells are capable of producing IL-22. In addition, myeloid cells such as macrophages, neutrophils and mast cells, as well as non-hematopoietic fibroblasts have been reported to have the ability to produce IL-22 in different disease models (Lanfranca et al., 2016).

Mammalian IL-22 signals through a heterodimeric receptor containing IL-22R1 and IL-10R2, with the latter also a component of the heterodimeric receptors for IL-10, IL-26, IL-28 and IL-29. IL-22 binds IL-22R1 that enables secondary binding of IL-10R2, thereby activating the receptor associated Jak1/Tyk2 kinases, leading to phosphorylation of these receptors and STAT proteins. In addition to Jak/STAT signalling, IL-22 receptor binding also activates the MAP kinase and p38 pathways (Dudakov et al., 2015). Whilst IL-10R2 is constitutively expressed in cells throughout the body and is a receptor subunit of several other IL-10 family members, IL-22R1 is a specific subunit for only IL-22 and is expressed almost exclusively in non-hematopoietic cells including epithelial cells, fibroblasts and intestinal stem cells in the gastrointestinal tract (Zenewicz, 2018). This allows directional signalling from immune cells that produce IL-22 to non-hematopoietic cells in tissues, especially at mucosal surfaces (Jones et al., 2008; Lanfranca et al., 2016).

IL-22 is a critical cytokine in the modulation of tissue responses during inflammation. It is upregulated in many chronic inflammatory diseases including psoriasis, rheumatoid arthritis and inflammatory bowel disease (Zenewicz, 2018). IL-22- and IL-22R1-deficient mice revealed a dual-natured role of IL-22 that can be protective or pathogenic (Eyerich et al., 2017). IL-22-mediated protection and regeneration of epithelial tissues has been well documented in multiple disease models via promotion of proliferation, inhibition of apoptosis and induction of antimicrobial molecules, including β-defensins and mucins, as well as proinflammatory cytokines such as IL-1, IL-6, IL-8, IL-11, G-CSF and GM-CSF (Dudakov et al., 2015). However, in a chronic setting IL-22-mediated proliferation and inhibition of apoptosis can lead to pathology such as malignancy and psoriasis (Zenewicz, 2018). Given its widespread action in regeneration, host defense and pathology, IL-22 is an attractive target for clinical development, making it one of the best-studied members of the IL-10 family of cytokines (Dudakov et al., 2015).

True orthologues of mammalian IL-10, IL-22 and IL-26 are present in teleost fish (Igawa et al., 2006; Secombes et al., 2011). A cytokine homologue related to mammalian IL-19/IL-20/IL-24 genes, termed IL-20 like (IL-20 L) has also been identified in fish (Wang et al., 2010). IL-22 has been cloned in many fish species, including fugu (*Takifugu rubripes*) (Zou et al., 2003), zebrafish (*Danio rerio*) (Igawa et al., 2006), Atlantic cod (*Gadus morhua*), haddock (*Melanograus aeglefinus*) (Corripio-Miyar et al., 2009), rainbow trout (*Oncorhynchus mykiss*) (Monte et al., 2011), turbot (*Scophthalmus maximus*) (Costa et al., 2012), so-iny mullet (*Lisa haematocheila*) (Qi et al., 2015), golden pompano (*Tranchinotus ovatus*) (Peng et al., 2017), yellow catfish (*Pelteobagrus filvidraco*) (Jiang et al., 2018) and mandarin fish (*Siniperca chuatsi*) (Huo et al., 2019). Fish IL-22 transcripts are highly expressed in mucosal tissues such as gills, intestine, fins and skin, and can be induced by PAMPs and bacteria (Wang and Secombes, 2013). For example, IL-22 was found to be induced *in vivo* by bacterial infections in rainbow trout (Harun et al., 2011; Monte et al., 2011; Chettri et al., 2012), turbot (Costa et al., 2012), pompano (Peng et al., 2017) and catfish (Jiang et al., 2018), by vaccination (Veenstra et al., 2017), and by stimulation with PAMPs and recombinant cytokines (IL-1β and TNFα) (Veenstra et al., 2018; Wangkahart et al., 2019b). Interestingly, IL-22 expression was highly induced in the gills of vaccinated and protected fish challenged with a lethal dose of bacteria, in haddock and rainbow trout (Corripio-Miyar et al., 2009; Harun et al., 2011). *In vitro* rainbow trout IL-22 transcripts can be induced in splenocytes by PMA and PHA (Monte et al., 2011), in head kidney (HK) cells by IL-21 (Wang et al., 2010), and in gut-associated lymphoid cells by PAMPs (LPS, flagellin and poly I:C) and recombinant cytokines (Attaya et al., 2018).

The recombinant IL-22 protein has been made and bioactivity studied in a few teleost fish species. Teleost IL-22 up-regulates the expression of antimicrobial peptide (AMP) genes (eg β-defensins, hepcidin and liver expressed antimicrobial peptide 2) (Monte et al., 2011; Costa et al., 2013; Huo et al., 2019) and administration of IL-22 significantly improves fish survival after bacterial challenge, as seen in turbot (Costa et al., 2013) and mullet (Qi et al., 2015). In contrast, knockdown of IL-22 in zebrafish increased pro-inflammatory cytokine expression in bacteria-stimulated fish and resulted in higher mortality after *Aeromonas hydrophila* infection (Costa et al., 2013). Such functional analysis suggests that IL-22 might have an important role in mucosal immunity in fish as seen in mammals and likely plays a major role in co-ordinating immune defence against bacterial pathogens and in vaccine-mediated immunity.

In common with most fish cytokines, little is known about IL-22 expression and modulation at the protein level in fish. Hence, in this study, we first produced monoclonal antibodies (mAb) against rainbow trout IL-22 that could specifically detect the recombinant and native IL-22 protein by Western blotting. We next studied the numbers of IL-22 positive cells by flow cytometry, and found that their numbers increase following stimulation of peripheral blood leucocytes (PBL) *in vitro* with killed bacteria, PHA and IL-21, and *in vivo* in blood and gills after bacterial infection. Lastly, immunohistochemistry revealed that IL-22 positive staining was found in epithelial cells within the gill filaments and cells in the interbranchial lymphoid tissue (ILT), suggesting that epithelial cells and lymphoid cells are important producers of IL-22 in fish gills.

## 2 Materials and methods

### 2.1 Fish

Juvenile rainbow trout were purchased from College Mill Trout Farm (Perthshire, UK) and maintained at 14 °C as described previously (Wangkahart et al., 2019a). Fish were fed twice daily on a commercial pellet diet (EWOS) and were given at least 2 weeks of acclimatization prior to any experimentation. All the experiments described comply with the Guidelines of the European Union council (2010/63/EU) for the use of laboratory animals and were carried out under UK Home Office project license PPL 70/8071, approved by the ethics committee at the University of Aberdeen.

### 2.2 IL-22 monoclonal antibody production

Two peptides, L7 (KEDLARVSRD) and L8 (TFLKDFCVHA) (Fig. S1), were predicted as being linear, accessible, hydrophilic, antigenic and present in low complexity regions, and located on the surface of native rainbow trout IL-22 using the Immune epitope database (IEDB) analysis resource software (https://www.iedb.org/home_v3.php). These candidate peptides were also subjected to Basic local alignment search tool (BLAST) (https://blast.ncbi.nlm.nih.gov/Blast.cgi) analysis against the salmonid proteome to ensure uniqueness to the target of interest, to reduce the potential for cross-reactivity and non-specific binding. The peptides selected were then synthesised by Almac Sciences Ltd and conjugated to ovalbumin (OVA) as carrier for immunisation and to bovine serum albumin (BSA) for screening. The procedure for generating the mAbs was as described previously (Alnabulsi et al., 2019). Resulting hybridoma clones were screened for specific antibody by ELISA using the relevant BSA-conjugated peptide as an antigen. Hybridoma clones that were strongly positive by ELISA were subcloned to ensure monoclonality. mAbs were isotyped using an Isostrip kit (Roche Diagnostics, UK) according to the manufacturer's instructions. The positive hybridoma clones were grown in Hybridoma-SFM (Thermo Fisher Scientific, UK) to produce working stocks of antibodies. The anti-IL-22 mAb-containing cell culture supernatants were stored at −20 °C until required. Antibody purification was performed by affinity chromatography using Prosep Ultra Affinity Chromatography Media (Millipore). The eluted IgG fractions were loaded onto a SDS-PAGE gel with known concentrations of BSA. Two protein bands were apparent corresponding to the heavy chains (~50 kDa) and light chains (~25 kDa) of IgG, respectively (Fig. S2). The most concentrated elution fraction was selected and diluted appropriately for experimentation. The IgG subclasses and light chain usage were determined using an IsoStrip kit (Roche, UK). Results showed that both mAbs against L7 and L8 are of the IgG1 isotype, with kappa light chains.

### 2.3 Detection of recombinant and endogenous IL-22 by Western blotting

Recombinant (r) IL-22 (Monte et al., 2011) and rIL-2B (Wang et al., 2018) used for Western blot analysis were prepared as described previously. 100 ng recombinant proteins were mixed with NuPAGE LDS loading buffer (Novex, Invitrogen) containing 5% β-mercaptoethanol (β-ME, Sigma-Aldrich) and incubated at 95 °C for 10 min before loading on a 4–12% Bis-tris SDS-PAGE gel (Invitrogen). The gel was run at 150 V for 1 h. Next the separated proteins were transferred onto a PVDF membrane (Millipore, USA) using an Xcell SureLock™ Electrophoresis Cell system (Invitrogen). The membranes were stained with SimplyBlue™ SafeStain (Life Technologies, UK) as per the manufacturer's instructions to confirm equal loading. The membrane was blocked with 5% milk-PBST (1X PBS containing 0.1% Tween-20), washed thrice with PBST and incubated with anti-IL-22 mAb L7 or L8 overnight at 4 °C. After washing and incubation with a secondary anti-mouse IgG peroxidase conjugated antibody (Sigma-Aldrich) (diluted 1 in 3000), the peroxidase activity was detected using a Supersignal West Pico Kit (Thermo Fisher Scientific,UK). The membrane was then exposed to X-ray film for 0.5–5 min and processed using Carestream Kodak autoradiography GBX developer (Sigma-Aldrich).

Rainbow trout IL-22 transcript expression can be induced in head kidney (HK) cells by rIL-21 (Wang et al., 2011). Thus, the ability of anti-IL-22 mAbs to detect endogenous IL-22 protein was tested in lysates of HK cells stimulated with rIL-21. The HK cells were prepared as described previously and resuspended at 2 × 10^6^ cells/ml in complete cell culture medium; Leibovitz L-15 medium (L-15, Gibco) supplemented with 100 IU/ml penicillin, 100 μg/ml streptomycin and 10% foetal calf serum (FCS, Lab Tech, UK). The resulting HK cells were stimulated with 100 ng/ml of rIL-21 protein (Wang et al. 2011), or left unstimulated as control, at 20 °C for 3 days. To increase intracellular retention of the expressed IL-22 protein, 1 μl of BD GolgiPlug™ containing brefeldin A (BD Biosciences, UK) was added for every 1 ml of cell culture medium 6 h before cell harvest in LDS loading buffer (Thermo Fisher Scientific,UK). The endogenous IL-22 protein was detected by Western blotting as described above.

### 2.4 Detection of IL-22 expression in PBL by intracellular staining

Rainbow trout peripheral blood leucocytes (PBL) were prepared by hypotonic disruption of erythrocytes as detailed previously (Hu et al., 2018) and resuspended to 2 × 10^6^ cells/ml in complete L-15 cell culture medium. Freshly prepared PBL were seeded into 12-well cell culture plates (Greiner bio-one, UK) at 2 ml/well, and stimulated with phytohemagglutinin from red kidney bean *Phaseolus vulgaris* (PHA, 5 μg/ml, Sigma-Aldrich), rIL21 (200 ng/ml), and a formalin killed bacterin of *Aeromonas salmonicida* subsp. *salmonicida* MT423 cultured under iron depleted conditions (100 μg/ml, Attaya et al., 2019) for 24 h. These stimulation conditions are known to increase trout IL-22 transcript expression (Monte et al., 2011; Wang et al., 2011; Attaya et al., 2019). BD GolgiPlug™ containing brefeldin A (BD Biosciences, UK) was added 6 h before cell harvest to allow the accumulation of intracellular cytokines. Both non-adherent and adherent cells were harvested, the latter using 0.5% trypsin-EDTA (GIBCO), for intracellular staining and flow cytometry analysis.

The live and dead cells were distinguished by Zombie staining using a Zombie Green™ Fixable Viability kit (Biolegend). Zombie Green™ dye was reconstituted in 100 μl DMSO following the manufacturer's guidelines and diluted at a ratio of 1:100 in flow cytometry (FACS) buffer; Dulbecco's phosphate buffered saline (Gibco) supplemented with 2% FCS. 1 × 10^6^ cells were re-suspended in Zombie green dye solution in the dark and incubated on ice for 20 min. The cells were kept in the dark by covering with foil in later procedures. The cells were washed twice with FACS buffer for 15 min on ice and fixed with fixation buffer (BD Cytofix solution containing 4.2% (W/V) paraformaldehyde, PFA, BD Biosciences, UK). The cells were again washed twice and permeabilised using a FACS buffer containing 0.1% (W/V) Saponin (Sigma-Aldrich) for 15 min on ice. The cells were then immunostained with anti-IL-22 mAb (10 μg/ml, clone L8) for 60 min on ice. After three washes with Saponin-FACS buffer, the cells were incubated with goat anti-mouse IgG1 Fc cross-adsorbed secondary antibody conjugated to APC (2 μg/ml, ThermoFisher Scientific) for a further 30 min. The cells were finally washed thrice with Saponin-FACS buffer and analysed using an Accuri C6™ Flow Cytometry (BD Biosciences) and FlowJo, LLC Single Cell Analysis software v10 (FlowJo LLC, USA). In all cases, isotype controls using a mAb against respiratory syncytial virus (RSV, clone 4.15, mouse IgG1, κ) were performed in parallel. The anti-RSV mAb was characterised previously (Gimenez et al., 1984) and has been used previously as an isotype control in rainbow trout, where no non-specific staining was found (Benedicenti et al., 2015).

### 2.5 IL-22 expression after bacterial infection

The causative pathogens of furunculosis (*A. salmonicida*) and enteric red-mouth disease (*Yersinia ruckeri*) were selected in this study due to their importance in salmonid aquaculture and known ability to induce IL-22 transcripts. The preparation of bacterial stocks and the infection procedure were as described previously (Harun et al., 2011; Pohl et al., 2018). Briefly, 6 rainbow trout (~200 g) were injected intraperitoneally (i.p.) with *A salmonicida* (Hooke strain) or *Y. ruckeri* (strain MT3072) at 1 × 10^6^ cfu in 0.5 ml PBS. A second group were i.p. injected with 0.5 ml PBS as control. Fish were killed by 2-phenoxyethanol (Sigma-Aldrich) over-dose at 24 h post-infection. Blood was withdrawn from the caudal vein and gill samples were taken from each fish. PBL were prepared as above. The gill samples from *A. salmonicida* infected fish were also used for RNA/protein expression and immunohistochemistry, in addition to gill leucocyte preparation for intracellular staining. A gill single cell suspension was obtained using an EASYstrainer (70 μm, Greiner Bio-One), and incomplete cell culture medium; L-15 medium supplemented with 10 IU/ml heparin (Sigma-Aldrich) and 2% FCS. The cell suspension was loaded onto 51% Percoll^®^ (Sigma-Aldrich) and centrifuged at 500×*g* for 40 min at 4 °C (without brake). The leucocytes at the interface were collected and washed twice with incomplete cell culture medium. The intracellular staining of both PBL and gill leucocytes was as described above.

### 2.6 Transcript and protein expression of IL-22 in the gills after *A. salmonicida* infection

Gill samples (50–100 mg) from *A. salmonicida* infected fish and the control fish were homogenised in 1.5 ml of TRI reagent (Sigma-Aldrich) using a Qiagen Tissue Lyser II, and stored at −80 °C until RNA/protein extraction. Total RNA was isolated following the manufacturer's guidelines. The cDNA synthesis and qPCR analysis of the expression of IL-22 and the house keeping gene EF-1α was carried out as described before (Wang et al., 2011). The IL-22 transcript expression was normalized to EF-1α and expressed as a fold change calculated as the average expression in infected samples divided by that in control samples.

The proteins in the organic phase (600 μl) after phase separation of the TRI lysate were precipitated with 900 μl isopropanol (Sigma Aldrich), washed thrice with 95% ethanol containing 0.3 M guanidine hydrochloride (Sigma-Aldrich) and once with 100% ethanol (Sigma-Aldrich). The pellet was dissolved in a buffer containing 0.5% SDS and 4 M urea in 10 mM Tris-HCl (pH 8.0) aided by sonication. The resultant protein samples were analysed by Western blotting for detection of IL-22 protein expression as above.

### 2.7 IL-22 expression in the gills after *A. salmonicida* infection detected by immunohistochemistry

Fresh gill samples from *A. salmonicida* infected trout were dissected, rinsed with 1X PBS (Sigma-Aldrich), and fixed with 4% PFA-PBS buffer for 18 h at 4 °C. The samples were next washed 5 times with PBS and stored in 70% ethanol at 4 °C. The wax embedding and sectioning of tissues were performed by the Microscopy and Histology Core Facility at the University of Aberdeen. Briefly, samples in 70% ethanol were loaded into a Citadel 2000 tissue processor (Fisher Scientific), washed using 70% ethanol for 2 h, 95% ethanol for 2 h, then with 100% ethanol for 2 × 3 h. The samples were then drained off until no residual alcohol was left, when chloroform (Honeywell)/xylene (Fischer chemicals) solution was added at a ratio of 1:1 for 2 h and replaced on two occasions. Finally, Cellwax (Cellpath) was added to the samples and left to set for 3 h, resulting in treated samples being embedded in a block of Cellwax. The Cellwax blocks were then cut into 5 μm sections with a Leica RM 2125 rotary microtome (Leica Biosystems) and mounted on to slides and stored at 4 °C until use.

Immunohistochemistry was performed using a Dako autostainer E 172566 (Model: LV-1, Dako universal staining system, Dako/Agilent Technologies LDA UK Limited, Cheadle, UK). Tissue sections were first dewaxed in xylene and rehydrated in decreasing ethanol concentrations and washed with tap water. The antigen retrieval was performed by microwaving the tissue sections for 20 min using a microwave (800 W) while sections were fully immersed in 10 mM citrate buffer (pH 6.0). After cooling, the slides were incubated with anti-IL-22 mA b (clone L8) for 60 min at room temperature and washed twice with Dako washing buffer. The endogenous peroxidase activity was blocked by incubation with hydrogen peroxidase blocking solution for 7 min (DAKO). The slides were then washed twice using Dako washing buffer and incubated with peroxidase-polymer labelled goat anti-mouse/rabbit secondary antibody (Dako EnVision™ FLEX Detection system, Cheadle, UK) for 30 min at room temperature. The peroxidase activity was revealed by incubation with the chromogen substrate 3, 3′-diaminobenzidine (DAB) for 7 min. Finally, the slides were submerged in Surgipath Harris Hematoxylin solution (Leica Biosystems) for 10 s to counterstain the cell nuclei, before being dehydrated in increasing concentrations of alcohol, then xylene prior to being mounted with a cover slip. As a negative control, anti-RSV mAb was used instead of anti-IL-22 mAb. Slides were also incubated with antibody diluent (Dako) instead of the primary antibody as a further negative control. Finally, sections were examined with a Zeiss Axioscop 40 light microscope and Zeiss AxioScan Z1 Slide scanner (Microscopy and Histology Core Facility, University of Aberdeen).

### 2.8 Statistical analysis

The data were analysed statistically using the SPSS Statistics package 24.0 (SPSS Inc., Chicago, Illinois). Real-time PCR data were scaled and log 2 transformed before statistical analysis, as described previously (Wang et al., 2011). Flow cytometry data were converted to the percentage of IL-22^+^ cells, then ARCSINE transformed for statistical analysis using either a paired-sample T-test or independent-sample T-test, with P ≤ 0.05 considered significant.

## 3 Results

### 3.1 Anti-trout IL-22 mAbs detect recombinant and native IL-22 by Western blotting

Screening of hybridoma supernatants against BSA-conjugated peptide by ELISA identified clones that reacted specifically to their immunogen. These clones were re-cloned and supernatant re-tested. The mAb L7 (against peptide L7, Fig. S1) reacted specifically with peptide L7 and rIL-22 but not with peptide L8 and rIFN-γ (Fig. S3). Similarly, mAb L8 reacted specifically with peptide L8 and rIL-22 but not peptide L7 and rIFN-γ (Fig. S3), indicating that both mAbs can react with trout rIL-22 in ELISA. The mAbs were purified for further analysis (Fig. S2).

**Fig. 1:**
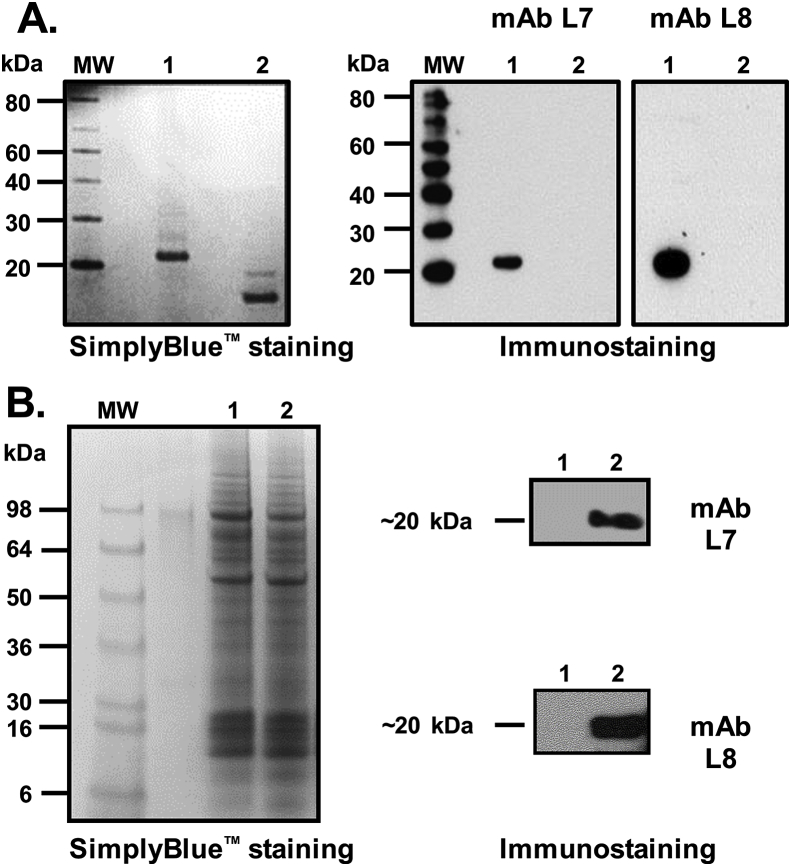
**Western blot detection of recombinant (A) and endogenous rainbow trout IL-22 (B).** (**A**) Recombinant IL-22 (lane 1, 100 ng) and rIL-2B (lane 2, 100 ng, Wang et al., 2018) proteins were fractionated in a 4–12% (w/v) Bis-Tris SDS-PAGE under reducing conditions, stained with SimplyBlue^™^ SafeStain (left) and immunostained with anti-trout IL-22 mAb L7 or L8 (right). MW = Magic-Marker™ XP Western Protein standard (Invitrogen). (**B**) Cell lysates from head kidney leucocytes cultured without (lane 1) or with rIL-21 (lane 2) were fractionated in a 4–12% (w/v) Bis-Tris SDS-PAGE under reducing conditions, stained with SimplyBlue (left) and immunostained with anti-trout IL-22 mAb L7 or L8 (right).

Both mAbs L7 and L8 reacted with rIL-22 in Western blots but not with the un-related protein rIL-2B, with both proteins possessing a his-tag (Fig. 1A). Furthermore, both mAbs detected no bands in un-stimulated controls but a protein of the expected size was seen with lysates of HK cells stimulated with rIL-21 that is a known inducer of IL-22 transcript expression (Wang et al., 2011) (Fig. 1B). These results demonstrated that the generated anti-IL-22 mAbs L7 and L8 can specifically detect both recombinant and native IL-22 protein by Western blotting.

### 3.2 Anti-trout IL-22 mA b detects IL-22-producing cells by intracellular staining

**Fig. 2:**
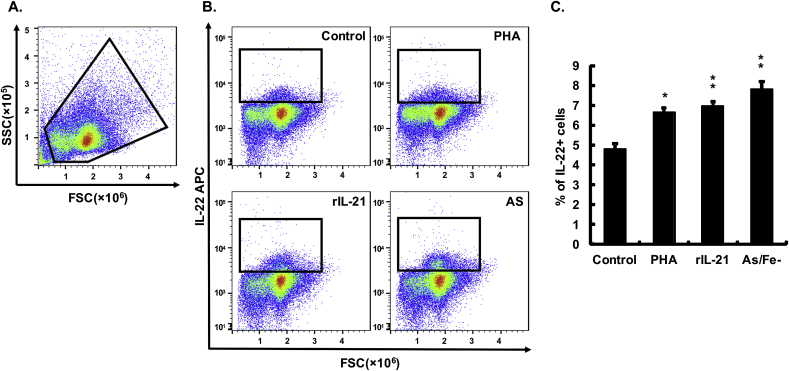
**Detection of IL-22 producing cells in PBL.** Freshly prepared PBL from four fish were individually stimulated with PHA (5 μg/ml), rIL-21 (200 ng/ml) and *A. salmonicida* (AS/Fe-, 100 μg/ml) or PBS only, as control, for 24 h. BD GolgiPlug™ containing brefeldin A was added 6 h before intracellular staining as described in the Materials and Methods. The IL-22^+^ cells were analysed using flow cytometry, with representative results from a single fish shown. (**A**) Gating of the leucocyte population. (**B**) Gating of IL-22^+^ cells in PBL stimulated with PHA, rIL-21, *A. salmonicida* bacterin (AS) and unstimulated controls. The isotope control staining is provided in Fig. S5. (**C**) Histogram showing the mean +SE percentage of IL-22^+^ cells in each treatment. A paired samples T-test analysis between stimulated and control samples is shown above the bars as *p ≤ 0.05, **p ≤ 0.01.

To investigate if IL-22 producing cells could be identified using the mAbs developed, intracellular immunostaining was performed using PBL following incubation with known IL-22 inducing stimulants. PBL were chosen for study over other immune cell populations due to the ease of preparing large amounts of high purity leucocytes using our recently described hypotonic method (Hu et al., 2018). Initial analysis suggested both mAb L7 and L8 stained a small percentage of unstimulated PBL but with L8 giving more consistent results, hence L8 was used for all subsequent intracellular staining. A standard procedure for gating was adopted as shown in Fig. S4, to eliminate any potential artifacts for data analysis. Cells were first analysed according to their FSC-A/SSC-A profile, with total leucocytes defined as those gated in Fig. 2. Cells were then analysed according to their FSC-A/FSC-H profile to exclude doublets, and dead cells were excluded following Zombie staining. Isotype controls for mouse mAbs were also tested in parallel to discern any non-specific binding of the Abs (Fig. S5). The proportion of IL-22^+^ cells was then calculated according to the isotype control staining, and revealed that in control PBL cultured for 24 h *in vitro* 4.77 ± 0.28% of the cells within the total leucocyte gate were IL-22^+^ (Fig. 2). This percentage was significantly increased to 6.65 ± 0.20%, 6.95 ± 0.23 and 7.80 ± 0.40% by stimulation with PHA, rIL-21 and the *A. salmonicida* bacterin, respectively. Taken together, these results show that the native IL-22 protein can be detected by intracellular staining of trout leucocytes using mAb L8.

### 3.3 IL-22 transcripts and IL-22 producing cells were increased in gills and PBL after bacterial infection

**Fig. 3:**
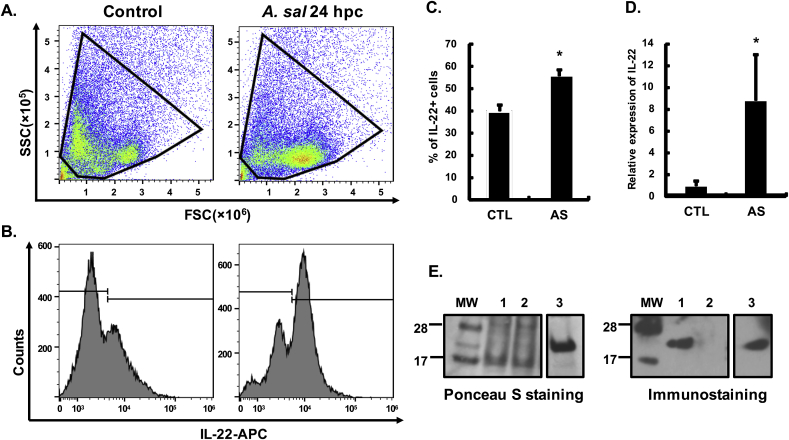
**IL-22 expression in the gills at 24 h after *A. salmonicida* infection.** Rainbow trout were infected by i. p. injection of *A. salmonicida* (AS), or PBS as control. Gill samples were collected 24 h post-challenge (hpc) for the preparation of leucocytes, total RNA and protein. Gill leucocytes were immunostained with anti-IL-22 mAb L8 and analysed by flow cytometry. Representative results are shown in (**A**) Gating of the leucocyte populations, and (**B**) Gating of IL-22^+^ cells. The isotope control staining is provided in Fig. S6A. (**C**) The percentage of IL-22^+^ cells in total leucocytes (mean +SE, N = 6). (**D**) Relative expression of gill IL-22 transcripts quantified by RT-qPCR (mean +SE, N = 6). The relative expression was firstly normalized against EF-1α, and presented as fold change calculated as the average expression level of infected fish divided by that of the control fish. * indicates a significant difference between control and infected fish (p ≤ 0.05, independent samples T-test). (**E**) Western blot detection of IL-22 protein in gill lysate. Total proteins were prepared from gill lysates of *A. salmonicida* infected or PBS-injected (as control) fish, and immunostained with anti-IL-22 mAb L8 (right) as described in Fig. 1. rIL-22 was used as a positive control. Representative results are shown. Ponceau S staining (left) indicated equal loading and efficient transferring of proteins to a PVDC membrane. Lane 1, 24 h post infection of *A. salmonicida*, Lane 2, control fish, Lane 3, rIL-22 protein.

We next examined the IL-22 producing cells *in vivo* after *A. salmonicida* infection. Gill leucocytes were immunostained intracellularly using the anti-IL-22 mAb L8, and analysed by flow cytometry. Total leucocyte gating was determined according to the live/dead cell Zombie staining and mouse IgG_1_ isotype control staining (Fig. 3A). Remarkably 39.88 ± 2.67% of leucocytes from control fish were IL-22^+^. The IL-22^+^ cells were significantly increased to 55.98 ± 2.69% in gill leucocytes at 24 h after *A. salmonicida* infection (Fig. 3B and C). Furthermore, IL-22 transcript levels were also significantly increased in the gill samples after infection (Fig. 3D). IL-22 protein was also detected by Western blotting in the gill lysates in *A. salmonicida* infected fish but not in control fish (Fig. 3E). Taken as a whole, IL-22 was shown to be increased at both the transcript and protein levels along with an increase of IL-22 producing cells in the gills after *A. salmonicida* infection.

**Fig. 4:**
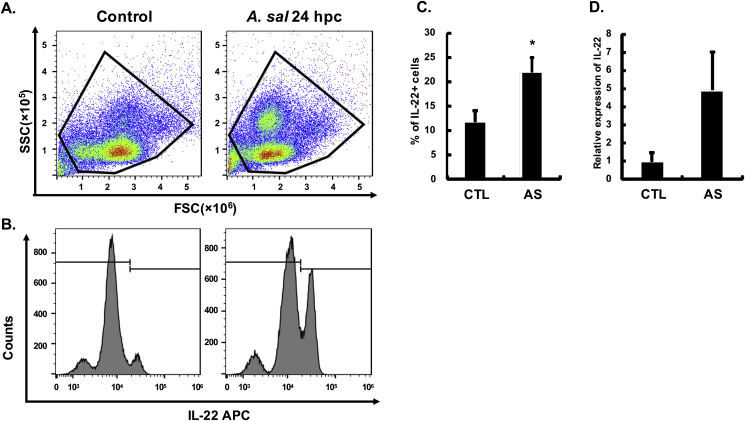
**IL-22 expression in PBL at 24 h after *A. salmonicida* infection.** Rainbow trout were infected by i. p. injection of *A. salmonicida* (AS), or PBS as control. Blood samples were collected 24 h post challenge (hpc) and PBL prepared. PBL were immunostained with anti-IL-22 mAb L8 and analysed by flow cytometry, or used for total RNA preparation for RT-qPCR analysis of IL-22 transcript expression. Representative results are shown in (**A**) Gating of the leucocyte population and (**B**) Gating of IL-22^+^ cells. The isotope control staining is provided in Fig. S6B. (**C**) The percentage of IL-22^+^ cells in total leucocytes (mean +SE, N = 6). (**D**) Relative expression of IL-22 transcripts in PBL quantified by RT-qPCR (mean +SE, N = 6). The relative expression was first normalized against EF-1α, and presented as a fold change calculated as the average expression level of infected fish divided by that of the control fish. * indicates a significant difference between control and infected fish (p ≤ 0.05, independent samples T-test).

Similarly, IL-22 producing cells (Fig. 4A–C) and IL-22 transcripts (Fig. 4D) were also increased in PBL 24 h after *A. salmonicida* infection. 11.86 ± 2.14% of leucocytes were IL-22^+^ in control fish, increasing to 21.96 ± 3.02% in infected fish.

**Fig. 5:**
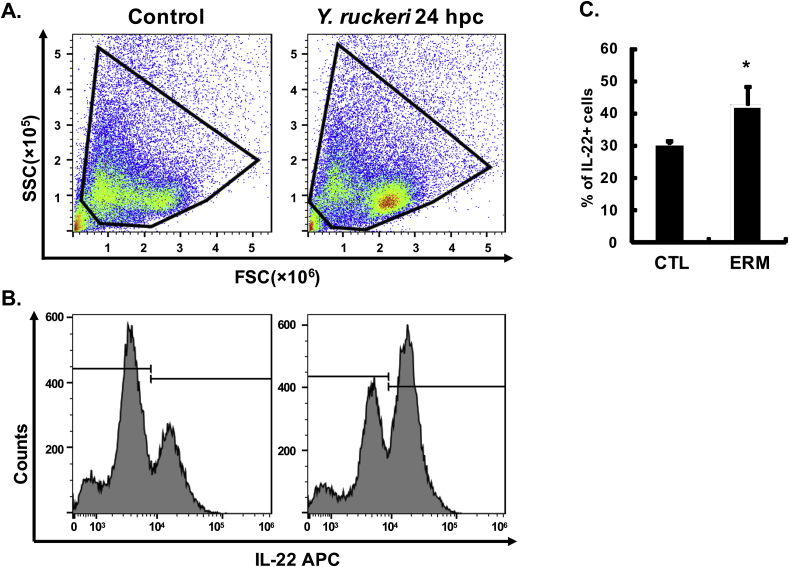
**IL-22-producing cells in gill leucocytes after *Y. ruckeri* infection.** Rainbow trout were infected by i. p. injection of *Y. ruckeri* (ERM) or PBS as control. Gill samples were collected at 24 h post challenge (hpc) and leucocytes prepared. Gill leucocytes were immunostained with anti-IL-22 mAb L8 and analysed by flow cytometry. Representative results are shown in (**A**) Gating of the leucocyte population and (**B**) Gating of IL-22^+^ cells. (**C**) The percentage of IL-22^+^ cells in total leucocytes (mean +SE, N = 6). * indicates a significant difference between control and infected fish (p ≤ 0.05, independent samples T-test).

**Fig. 6:**
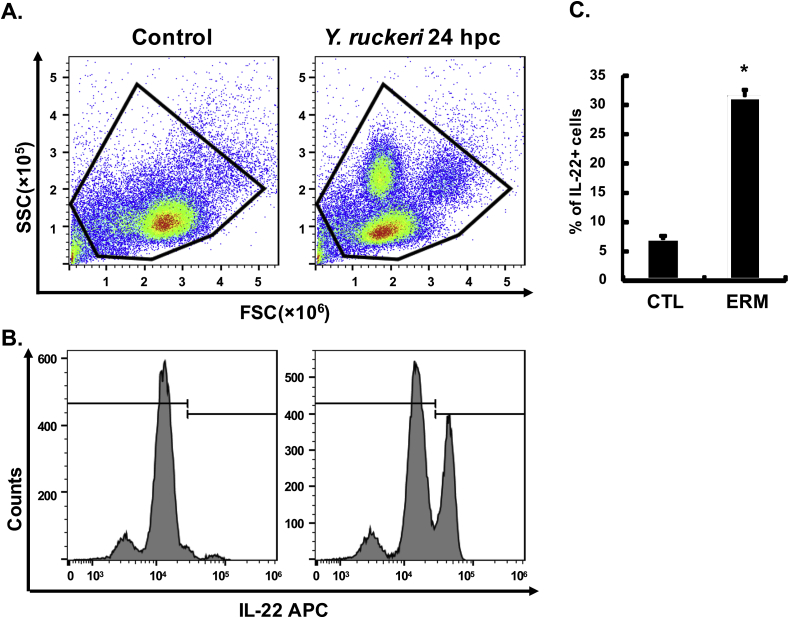
**IL-22-producing cells in PBL after *Y. ruckeri* infection.** Rainbow trout were infected by i. p. injection of *Y. ruckeri* (ERM) or PBS as control. Blood samples were withdrawn at 24 h post challenge (hpc) and PBL prepared. PBL were immunostained with anti-IL-22 mAb L8 and analysed by flow cytometry. Representative results are shown in (**A**) Gating of the leucocyte population and (**B**) Gating of IL-22^+^ cells. (**C**) The percentage of IL-22^+^ cells in total leucocytes (mean +SE, N = 6). * indicates a significant difference between control and infected fish (p ≤ 0.05, independent samples T-test).

The IL-22 producing cells induced by bacterial infection was also examined in another important salmonid disease model, yersiniosis caused by *Y. ruckeri*, in a separate experiment. In gill leucocytes, 30.50 ± 0.85% were IL-22^+^ in control fish and this was increased significantly to 45.35 ± 5.84% at 24 h after *Y. ruckeri* infection (Fig. 5). In PBL, 6.98 ± 0.63% were IL-22^+^ in control fish and this increased to 31.35 ± 1.17% at 24 h after *Y. ruckeri* infection (Fig. 6).

### 3.4 Rainbow trout IL-22 is expressed in interbranchial lymphoid tissue and gill epithelial cells

**Fig. 7:**
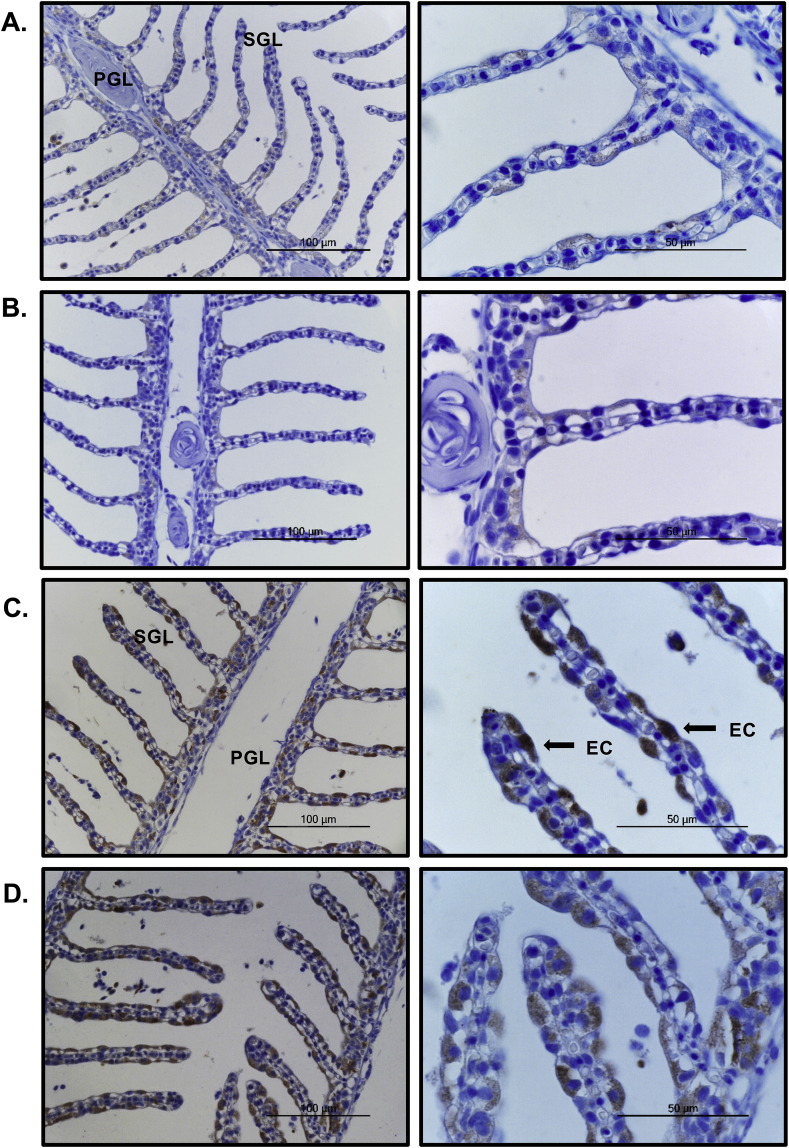
**IL-22 producing cells in the gills.** Rainbow trout were infected by i. p. injection of *A. salmonicida* or PBS as control. Gill samples were collected for detection of IL-22 producing cells by immunohistochemistry using anti-IL-22 mAb L8. Each slide was counter-stained with hematoxylin (HE). Primary gill lamellae (PGL) and secondary gill lamellae (SGL) are shown. Black arrow heads indicate IL-22^+^ staining. Representative images from two control fish or two infected fish are shown at 40× magnification (left images, scale bars = 100 μm) and at 100× magnification (right images, scale bars = 50 μm). **(A, B)** Relatively few, weakly positive cells were observed in the control fish 24 h post injection. **(C, D)** Intense staining was seen in the epithelium of the PGL and SGL of *A. salmonicida* challenged fish.

Immunohistochemistry analysis for rainbow trout IL-22 was performed to visualise the IL-22 positive cells in gills post *A. salmonicida* infection. For consistency of data analysis, the second gill arch from the left gill cavity was taken from each fish 24 h after *A. salmonicida* infection or PBS-injection. Horizontal sections of the middle gill arch containing gill raker, primary gill lamellae (PGL) and secondary gill lamellae (SGL) were examined. Weakly IL-22^+^ cells were found in the control fish gills, mostly located in the epithelium of the PGL and SGL (Fig. 7A–B). Intense IL-22 positive staining was found in the gill epithelium of *A. salmonicida* infected fish (Fig. 7C–D). The signal was observed mainly in the differentiated epithelial cells that have contact with the external environment, and in some cells also in the undifferentiated basal cells inside the epithelium. Common types of differentiated epithelial cells are squamous or cuboidal pavement cells (PEs), mitochondria-rich cells (MRCs) and mucus-producing goblet cells. No signal was detected in any part of the gill in negative control staining.

**Fig. 8:**
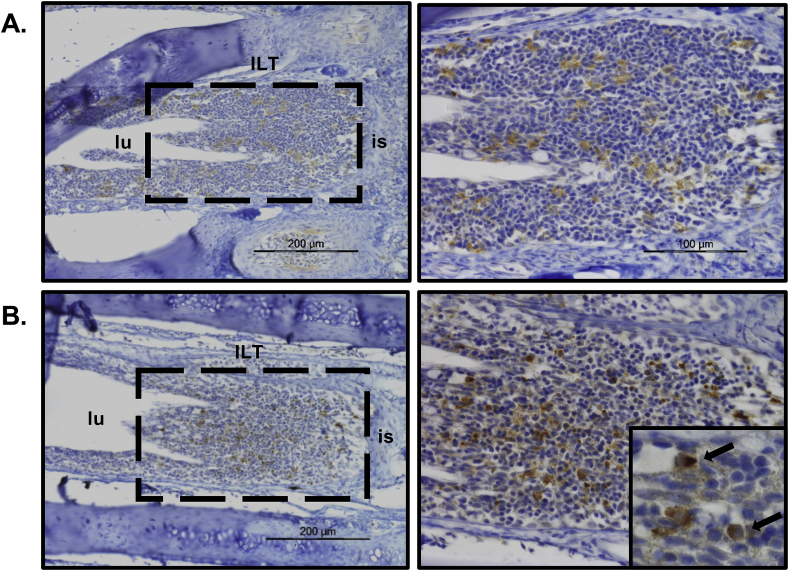
**Scattered IL-22 producing cells in gill interbranchial lymphoid tissue (ILT).** Rainbow trout were infected by i. p. injection of *A. salmonicida* or PBS as control. Gill samples were collected for detection of IL-22 producing cells by immunohistochemistry using anti-IL-22 mAb L8. Each slide was counter-stained with hematoxylin (HE). IL-22^+^ cells were present in the ILT between the lumen (lu) and interbranchial septum (is). **(A)** Relatively weak staining in ILT from control fish. **(B)** More intense staining in ILT from *A. salmonicida* infected fish. Inset shows an enlarged image with IL-22^+^ lymphoid-like cells.

The interbranchial lymphoid tissue (ILT) in salmonids is located at the terminal portion of the interbranchial gill septa, as a distinct structure that contains T cells and antigen-presenting cells embedded in a meshwork of epithelial cells interconnected by desmosomes (Koppang et al., 2010, 2015). Following immunohistochemistry with mAb L8, IL-22^+^ cells were found in the ILT both in control and *A. salmonicida* infected fish, with the latter showing more intense cytoplasmic staining (Fig. 8).

## 4 Discussion

Interleukin-22 is a critical cytokine in modulating tissue responses during inflammation. It is one of the most studied members of the IL-10 family, and is produced mainly by CD4^+^ T helper cells, NKT cells and type 3 innate lymphoid cells (ILC). In mammals, IL-22 is recognised to increase during chronic inflammatory diseases, preventing tissue damage during immune responses (Zenewicz et al., 2007). It also has an important antimicrobial function by enhancing the expression of antimicrobial peptides in non-hematopoietic cells, including keratinocytes and epithelial cells, with the IL-22 receptor showing a remarkable tissue/cell-specificity (Aujla et al., 2008; Zheng et al., 2008; Liang et al., 2006). In fish, IL-22 appears to have a similar biological function to mammals, and can modulate AMP expression suggesting a potent role in triggering antimicrobial defences for microbial clearance (Monte et al., 2011). IL-22 is also an important indicator of Th17/Th22 responses in mammals. Whilst equivalent responses are still to be confirmed in fish the coordination of the innate and adaptive immune system is believed to be mediated by T helper cell subsets (Dee et al., 2016; Takizawa et al., 2016). Nevertheless very little is known about the cell types that produce IL-22 in fish.

Due to a lack of suitable tools to recognise immune molecules at the protein level, studies of fish cytokines have been hindered. To find out more about the function of IL-22 in fish, in terms of detecting the secreted protein and the types and numbers of cells producing this cytokine, we have used a synthetic peptide immunisation approach to generate two rainbow trout anti-IL-22 mAbs (L7 and L8) to allow such studies. These mAbs were fully validated, by ELISA against their peptide immunogens and with Western blots that showed they specifically recognise recombinant trout IL-22 produced in *E. coli* and the native IL-22 protein in rainbow trout following *in vitro* and *in vivo* induction (see below). Whilst only a single protein band was detected in Western blots, the size was a little larger than the predicted ~17 kDa mature trout IL-22 protein (Monte et al., 2011). Since there are no glycosylation sites in the mature peptide and since both the recombinant IL-22 and native IL-22 were detected at a similar size (with rIL-22 slightly larger due to the his-tag), this suggests that buffer effects and/or secondary structure affected the speed of migration in the gels.

Our previous studies have demonstrated that IL-22 transcript expression was induced significantly by rIL-21 stimulation of HK cells in culture (Wang et al., 2011), so we next investigated whether IL-22 protein expression was also increased in such cells. Since the IL-22 transcripts were increased maximally 24–72 h post-stimulation we used 24 h in this study. Our Western blot results clearly showed the induction of IL-22 protein, detectable using both mAbs. This both verified the ability of these mAbs to recognise native trout IL-22, as well as their specificity in that no other protein bands were detectable with the HK lysates. Since we have recently developed an efficient method of PBL isolation using hypotonic lysis (Hu et al., 2018), we used PBL and rIL-21 stimulation to see if we could also detect intracellular IL-22 in these cells by flow cytometry. In addition, we used PHA stimulation, as another known inducer of IL-22 transcripts *in vitro* (Monte et al., 2011) and killed *A. salmonicida* since live and killed *A. salmonicida* have been shown to induce IL-22 transcripts *in vivo* and *in vitro* respectively (Costa et al., 2012; Kumari et al., 2015). The flow cytometry data showed a small but detectable number of IL-22^+^ cells in PBL (~5%), that was increased significantly by the three stimulants, with the *A. salmonicida* bacterin giving the largest increase (to ~8%). Curiously the IL-22 transcript level was not increased by LPS in trout HK cells (Monte et al., 2011), so perhaps other components of the bacteria were stimulatory, potentially the IROMPS induced by the iron-depleted culture conditions used (Hirst and Ellis, 1994).

IL-22 transcript expression is detectable in most tissues but previous reports have shown that the expression of IL-22 in naïve fish is higher in mucosal tissues such as intestine and gills (Monte et al., 2011; Igawa et al., 2006; Costa et al., 2012; Peng et al., 2017; Jiang et al., 2018). Similarly following challenge with live bacteria IL-22 has been shown to increase markedly at these mucosal sites in naïve and vaccinated fish (Corripio-Miyar et al., 2009; Kumari et al., 2015; Jiang et al., 2018). These findings suggest that IL-22 may play a potentially important role in mucosal immunity against microbial diseases, as reported in mammalian studies, where IL-22 expression at mucosal sites is induced after bacterial infections (Aujla et al., 2008; Zheng et al., 2008). Indeed, our previous studies with trout and haddock have shown that IL-22 is increased in the gills of vaccinated fish that have been challenged with the appropriate pathogen, and is one of the few examples of a cytokine that has higher expression in vaccinated fish vs control fish (Corripio-Miyar et al., 2009; Harun et al., 2011). Typically, most pro-inflammatory cytokines are less inducible in vaccinated fish after challenge. Hence, in further analysis of IL-22^+^ cells in trout we focussed on PBL and gill cells, in fish challenged with two different bacterial pathogens previously shown (as outlined above) to induce IL-22 expression in fish, namely *A. salmonicida* and *Y. ruckeri*.

*A. salmonicida* is a Gram negative-bacterium that causes typical furunculosis and leads to significant losses to the salmonid aquaculture industry (Bernoth, 1997; Wiklund and Dalsgaard, 1998). Infected fish show acute or chronic features, such as septicaemia associated with multi-systemic haemorrhages or development of furuncles consisting of necrotic tissue in the skin. *A. salmonicida* enters the fish body from multiple sites including the skin, gills and intestine, and then rapidly diffuses to the internal organs leading to death of the infected fish (Farto et al., 2011; Dallaire-Dufresne et al., 2014). *Y. ruckeri* is also a Gram negative-bacterium, that is the causative agent of enteric red mouth disease in (mainly) salmonids, and causes significant losses in trout farming (Fernández et al., 2007; Tobback et al., 2007). Following infection with *A. salmonicida* for 24 h, both IL-22 transcript and protein expression were significantly induced in the gills. The results were confirmed by several techniques, including Western blotting, flow cytometry and immunohistochemistry (see below). Whilst the challenge was administered by i.p. injection, when fish are infected with bacteria the immune response is rapidly activated, with immune cells induced to secrete cytokines to enable the infected sites to resist pathogen invasion (Raida et al., 2011), and past studies comparing co-infection and i.p. injection of fish with *Y. ruckeri* have shown comparable induction of IL-22 in blood cells (Monte et al., 2016). Indeed, even injection of a *Y. ruckeri* vaccine bacterin by i.p. injection can induce AMP and acute phase protein expression in the gills shortly after injection (Wangkahart et al., 2019a). IL-22-producing cells were also increased in the PBL, which increased to a relatively high level (~30%) and with a very clear peak of IL-22^+^ cells seen by flow cytometry. These findings were in essence repeated using *Y. ruckeri* challenged fish, where again IL-22^+^ cells increased considerably upon infection. This remarkable increase of IL-22^+^ cells in PBL was accompanied by the visualisation of an addition population of cells in the FSC-A/SSC-A plots, that appeared to be myeloid cells and potentially were neutrophils. There are many precedents for the appearance of IL-22 producing neutrophils in mammals in disease states, as seen during intestinal inflammation/colitis (Zindl et al., 2013; Aden et al., 2016; Zhou et al., 2018), and with *Leishmania* infections (Carlsen et al., 2015). It is proposed that granulocytes have an important role in enhancing epithelial barrier function by release of granule-packaged IL-22 that may provide a more rapid release of this cytokine relative to what can be achieved by other innate lymphoid cells and lymphocytes (Zindl et al., 2013). Recent studies have even shown that acute phase proteins (eg. SAA3) can expand the numbers of IL-22 producing neutrophils (Zhang et al., 2018), and that this function is associated with expression of CD177 on their surface (Zhou et al., 2018). However, in the case of the trout gill cell preparations, it seems unlikely that the appearance of activated neutrophils would fully account for the increase in IL-22^+^ cells seen (ie via residual PBL in the preparations), especially as the IgM^+^ B cell numbers were consistently 6–10% in the gill cell suspensions compared to 45–60% in PBL (unpublished data). Nevertheless to confirm whether IL-22 expressing cells could be detected in gill tissue per se, we next undertook immunohistochemical analysis of samples taken from control and *A. salmonicida* infected trout to visualise where they were located, as outlined below.

A degree of IL-22 reactivity was seen in the gill lamellae and the ILT of control fish following immunohistochemistry of gill sections with mAb L8, and likely represents a baseline level of secretion to maintain the barrier (antimicrobial) function of the gill epithelial surface, as seen in mucosal tissues in mammals (Kumar et al., 2012; Moyat et al., 2016). Following *A. salmonicida* infection intensely stained IL-22^+^ cells were found in both locations (lamellae and ILT). These cells were not neutrophils, were not associated with the lamellar capillaries (the ILT is avascular – Aas et al., 2017), and appeared to be epithelial in nature in the lamellae but lymphoid in the ILT. As discussed above, in mammals IL-22 is produced by a variety of innate lymphoid cells and lymphocyte subpopulations (Cella et al., 2009; Witte et al., 2010). T cells are certainly present in the gills and ILT of fish, as identified with markers such as CD3, CD4 and CD8, and are quite numerous in the ILT but more scattered and relatively few in the branchial epithelium and lamellae (Koppang et al., 2010; Takizawa et al., 2011; Dee et al., 2016). The ILT is considered to be a secondary lymphoid tissue able to react to antigens, although its structure resembles that of the thymus (Aas et al., 2014). Hence IL-22 secreting cells at this site or IL-22^+^ cells migrating from this site into the gill would likely contribute to the local antimicrobial responses following infection. However, it is clear that at least another population of cells is present in the lamellae that also rapidly upregulate IL-22 expression in response to bacterial infection.

It is noteworthy that although IL-22^+^ cells were detected in gill leucocytes by intracellular staining, in interbranchial lymphoid tissue and gill epithelial cells by IHC, IL-22 protein was not detectable in gill lysate of control fish by Western blotting. This contradiction might be caused by two factors. First is the sensitivity (detection limit) of the method used. Western-blotting can detect a protein at the nanogram or sub-nanogram level in the whole sample that may be less sensitive compared to intracellular staining or IHC that detect the presence of IL-22 in a single cell that may have a relatively high concentration of IL-22. Second is that gill leucocytes and IL-22 expressing epithelial cells only represent a proportion of all gill cells present, thus the IL-22 expressed might be too diluted to be detectable in whole gill lysate by Western blotting.

Overall, we have generated two mAbs to rainbow trout IL-22, to gain an insight into the cells and sites of IL-22 production, as well as measuring more generally the secretion of IL-22 in this species. These mAbs have been shown to work in a variety of assays, from ELISA and Western blotting, to flow cytometry and immunohistochemistry. The focus here was on mucosal defences and whether IL-22 protein expression can be up-regulated in response to bacterial infection, and the analysis gives some intriguing results in terms of the variety of cell types that seem capable of producing IL-22 in fish.
