# Supplementary material for: Induction of IL-22 protein and IL-22-producing cells in rainbow trout Oncorhynchus mykiss
Source: Dev Comp Immunol. 2019 Dec;101:103449. doi: 10.1016/j.dci.2019.103449 (PMC6873780; doi:10.1016/j.dci.2019.103449)
Supplement: Multimedia component 1 [file mmc1.docx]

 **Fig. S1:** **The amino acid sequence of rainbow trout IL-22.** The peptide sequences used as immunogens to make the rainbow trout anti-IL22 monoclonal antibodies (mAbs) are highlighted in red (L7) and yellow (L8).

**Fig. S2: SDS-PAGE analysis of anti-IL22 monoclonal antibodies before and after purification.** Lane 1: BSA 5 µg, Lane 2: BSA 500 ng, Lane 3: unprocessed hybridoma culture supernatant L7, Lane 4: purified L7, Lane 5: unprocessed hybridoma culture supernatant L8, Lane 6: purified L8. All of the mAbs have two major protein bands at ~25 kDa and ~50 kDa in the denatured form, representing the light and heavy chains, respectively.

**Fig. S3:** **Reactivity of anti-IL-22 mAbs towards synthetic peptide immunogens and full-length recombinant IL-22 protein produced in *E. coli*.** Direct ELISA assays were performed using anti IL-22 L7 (A) and L8 (B) mAb. Columns represent the mean absorbance + SE values of 6 replicates (N=6). Both the L7 (A) and L8 (B) mAb were shown to specifically react with recombinant IL-22 and their associated peptide immunogens but not with recombinant IFNγ produced in a similar way as IL-22.


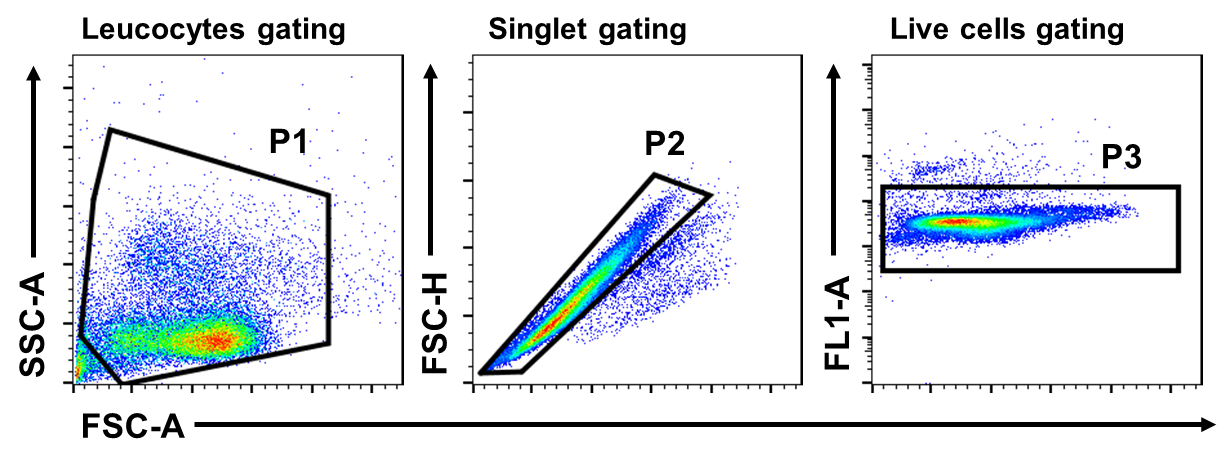


**Fig. S4: Gating strategy for flow cytometric analysis.** Examples for PBL purified by hypotonic lysis. P1 = Gate of valid leucocytes. P2 = Gate of singlets in FSC-H/FSC-A. P3 = Gate of live cells after Zombie Green^TM^ Fixable Viability staining detected by green fluorescence in FL1-A.

**
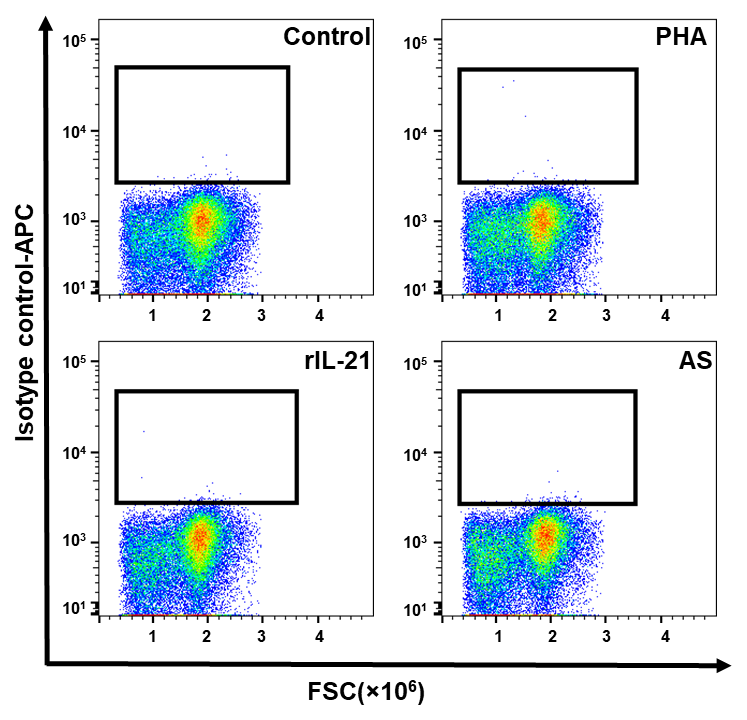
**

**Fig. S5. Isotype control staining of *in vitro* stimulated PBL.** Trout PBL stimulated with PHA (10 µg/ml), rIL-21 (200 ng/ml) and *A. salmonicida* (AS, 100 µg/ml) or PBS only for 24 h and immunostained with anti-RSV mAb as isotope control for the anti-IL-22 mAb.

**
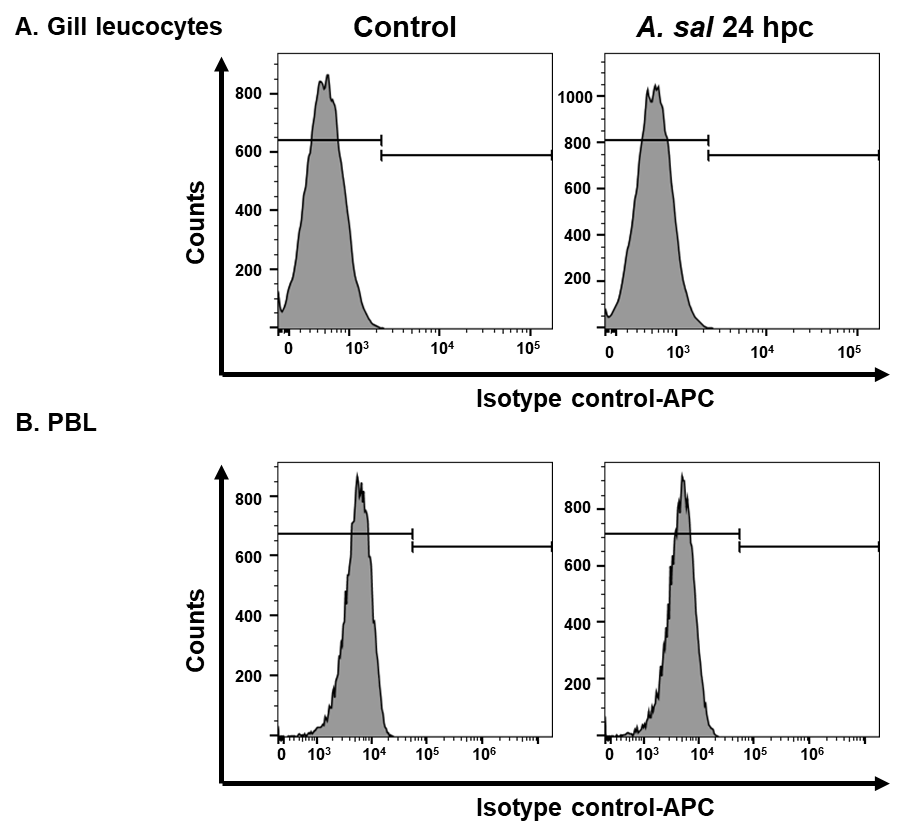
**

**Fig. S6. Isotype control staining of *in vivo* stimulated gill leucocytes and PBL.** Trout were infected by i.p. injection of *A. salmonicida*, or PBS for 24 h. Gill leucocytes and PBL were immunostained with anti-RSV mAb as isotope control of anti-IL-22 mAb.
